# Evaluating the use of synthetic T1-w images in new T2 lesion detection in multiple sclerosis

**DOI:** 10.3389/fnins.2022.954662

**Published:** 2022-09-29

**Authors:** Liliana Valencia, Albert Clèrigues, Sergi Valverde, Mostafa Salem, Arnau Oliver, Àlex Rovira, Xavier Lladó

**Affiliations:** ^1^Research Institute of Computer Vision and Robotics, University of Girona, Girona, Spain; ^2^Tensor Medical, Girona, Spain; ^3^Department of Computer Science, Faculty of Computers and Information, Assiut University, Asyut, Egypt; ^4^Magnetic Resonance Unit, Department of Radiology, Vall d'Hebron University Hospital, Barcelona, Spain

**Keywords:** brain, MRI, synthetic images, deep learning, multiple sclerosis

## Abstract

The assessment of disease activity using serial brain MRI scans is one of the most valuable strategies for monitoring treatment response in patients with multiple sclerosis (MS) receiving disease-modifying treatments. Recently, several deep learning approaches have been proposed to improve this analysis, obtaining a good trade-off between sensitivity and specificity, especially when using T1-w and T2-FLAIR images as inputs. However, the need to acquire two different types of images is time-consuming, costly and not always available in clinical practice. In this paper, we investigate an approach to generate synthetic T1-w images from T2-FLAIR images and subsequently analyse the impact of using original and synthetic T1-w images on the performance of a state-of-the-art approach for longitudinal MS lesion detection. We evaluate our approach on a dataset containing 136 images from MS patients, and 73 images with lesion activity (the appearance of new T2 lesions in follow-up scans). To evaluate the synthesis of the images, we analyse the structural similarity index metric and the median absolute error and obtain consistent results. To study the impact of synthetic T1-w images, we evaluate the performance of the new lesion detection approach when using (1) both T2-FLAIR and T1-w original images, (2) only T2-FLAIR images, and (3) both T2-FLAIR and synthetic T1-w images. Sensitivities of 0.75, 0.63, and 0.81, respectively, were obtained at the same false-positive rate (0.14) for all experiments. In addition, we also present the results obtained when using the data from the international MSSEG-2 challenge, showing also an improvement when including synthetic T1-w images. In conclusion, we show that the use of synthetic images can support the lack of data or even be used instead of the original image to homogenize the contrast of the different acquisitions in new T2 lesions detection algorithms.

## 1. Introduction

Artificial intelligence, particularly deep learning (DL), is currently widely used in medical imaging applications (Zhou et al., 2021; Chen et al., 2022). Tasks such as processing images (Razzak et al., 2018), segmenting anatomical structures (Fritscher et al., 2016) or diagnosing diseases such as stroke (Feng et al., 2018), brain tumors (Işın et al., 2016), and multiple sclerosis (Nair et al., 2020), are subjects of numerous domains of research. DL has been demonstrated to be a revolutionary tool in the field, improving state-of-the-art results. However, the algorithms developed with DL techniques have the major drawback of needing a large amount of data to train the model. Traditional data augmentation approaches, such as geometric transformations, intensity operations, filtering (Shorten and Khoshgoftaar, 2019), and deformable techniques such as deformable image registration or randomized displacement field, have been used to overcome this inconvenience. Nevertheless, some of these techniques have their own limitation such as the case of the geometric transformations which do not account for variations resulting from different imaging protocols or sequences, sizes, shapes, locations and appearances of the specific pathology (Yi et al., 2019) and produce highly correlated images in the training set, which prevents model improvements. Therefore, novel ways to mitigate these limitations have been studied including the use of image synthesis with DL (Chlap et al., 2021).

Image synthesis consists of the generation of new parametric images, including deriving more tissue contrast from a collection of image acquisitions (Lundervold and Lundervold, 2019). Image synthesis makes the synthesis of new medical images possible, including images that may not have been available in the original dataset. In medical imaging, image synthesis has been explored using different approaches, such as atlas based approaches (Burgos et al., 2015), machine learning approaches (Jog et al., 2017) and, lately, deep learning techniques (Pinaya et al., 2022), especially the use of generative adversarial networks (GANs) (Yi et al., 2019). This last method is currently widely used. The GAN framework was proposed by Goodfellow et al. (2014) and has lead to impressive results. Using GANs, it is possible to generate realistic- looking images from an implicit distribution that follows the real data distribution (Kazeminia et al., 2020). GAN approaches for synthesis can be either conditional, where an example of the desired output is specified and therefore labeled datasets are needed; or unconditional, where the output is a sample of a random class, using as unique input a noise vector. Unconditional strategies are less applied in the medical field. However, there were several studies, such as the one by Bermudez et al. (2018), where a deep convolutional GAN (DCGAN) learned to mimic the distribution of an entire high resolution magnetic resonance (MR) image, resulting in synthetic images that human observers could not reliably distinguish from the real images. From the conditional point of view, there are a large variety of works. For instance, in the image translation from computed tomography (CT) images to MR images, Wolterink et al. (2017) proposed a strategy using unpaired data of CT and MR cardiac images fed in a Cycle Consistency GAN (CycleGAN) (Zhu et al., 2017) for image translation and corresponding segmentation mask. The use of cross-modality in MR studies, such as the proposal by Lee et al. (2020), where a missing MR image (modality) can be inferred using its remaining contrast pairs with the application of collaGAN, an image imputation method (Lee et al., 2019). In Hi-Net (Zhou et al., 2020), the authors used different synthesis combinations, such as T1 and T2 sequences, to synthesize Fluid-attenuated inversion recovery (FLAIR) sequences, T1 and FLAIR sequences to synthesize T2 sequence, and T2 and FLAIR sequences to synthesize T1 sequences. Zhou et al. (2020) showed how their method outperformed state-of-the-art methods such as the pix2pix model (Isola et al., 2017) or CycleGAN (Zhu et al., 2017) by utilizing the correlation between different modalities for a modality-specific network that learns the representation of each individual modality and a fusion network dedicated to learn the common latent representation of the multimodal data.

Many medical image analysis approaches can take advantage of image synthesis as an strategy to overcome the lack of data or the necessity of several MR sequences. This is the case for multiple sclerosis (MS) which is a central nervous system inflammatory demyelinating disorder. MRI plays an essential role in establishing an accurate and early diagnosis of MS (Hemond and Bakshi, 2018), and monitoring treatment response, mainly by assessing new T2 lesion formations. There are several approaches of new T2 lesions detection pipelines using DL (McKinley et al., 2020; Salem et al., 2020). Two typical constraints in the pipelines are the lack of annotated data and the necessity of these models to use more than one MR image modality in order to determine the number, size and location of the lesion. Hence, some image synthesis proposals have been developed to overcome this drawback. For instance, Salem et al. (2019) proposed a model to generate synthetic MS lesions in MR images, while Wei et al. (2019) developed a model to synthesize the FLAIR modality by mapping multisequence source images.

We contribute to literature through the application of image synthesis to improve new T2 lesions detection for MS studies. To do so, synthetic T1-w MR images obtained of the original T2-FLAIR sequence are used in an algorithm for new T2 lesions detection. For the synthesis of the images, we propose an adversarial synthesis method based on the pix2pix approach (Isola et al., 2017). The performance of the synthetic images is evaluated when using them in the new T2 lesions detection pipeline from Salem et al. (2020). We also present the results of applying the proposed strategy to the MSSEG-2 challenge (Commowick et al., 2021). Our primary contribution is to demonstrate that the addition of synthetic T1-w images can contribute to the improvement of the sensitivity of the new T2 lesion detection algorithms when added to the original T2-FLAIR image as input to the detection models.

## 2. Materials and methods

In the development of this analysis, we used an in-house clinical dataset. The synthesis pipeline is based on 3D conditional GANs inspired by the pix2pix approach (Isola et al., 2017), while the recent proposal of Salem et al. (2020) is used for the detection of the new T2 lesions.

### 2.1. Dataset

The dataset used in this study contains 136 cases of MS patients with clinically isolated syndrome (CIS) where 73 cases had new T2 lesions in follow-up scans. The mean time between MR scans was 12 months (range 3–27 month). Basal and follow-up scans were obtained using a Siemens Tim Trio 3T with a 12-channel phased array coil. The MRI protocol included sagittal T1- weighted 3D magnetization-prepared rapid acquisition of gradient echo (MPRAGE) [repetition time (TR) = 2,300 ms, echo time (TE) = 2.98 ms, inversion time (TI) = 900 ms, voxel size = 1.0 x 1.0 x 1.2 *mm*^3^] and transverse fast fluid-attenuated inversion recovery (FLAIR) (TR = 5,000 ms, TE = 394 ms, TI = 1,800 ms, flip angle = 120°, voxel size = 1.0 x 1.0 x 1.0 *mm*^3^). The protocol was approved by the Vall d'Hebron Hospital (Barcelona, Spain) Research and Ethics Committee. Informed consent was obtained from each participant before enrolment in the study.

As the gold standard to evaluate the detection method, the number of new/enlarging T2 lesions was obtained after the review of the MRI images by an expert observer (a technician with more than 15 years of experience in assessing new T2 lesions for MS under neuroradiologist supervision) who was not blinded to the radiological report or clinical information.

In addition, we used the MSSEG-2 challenge dataset (Commowick et al., 2021) to extend the evaluation of our approach. A total of 100 MS patients were gathered where only 3D FLAIR sequences were acquired at a first and second timepoints (separated in from 1 to 3 years in time) using a total of 15 different MRI scanners (three GE scanners, six Philips scanners, and six Siemens scanners). The image characteristics vary with different resolutions and different voxel size (from 0.5 *mm*^3^ to 1.2 *mm*^3^). Data was separated according to 40 scans for training and 60 for testing. This database allows us to test the usefulness of our approach when missing T1 images in the training set.

### 2.2. Methodology

#### 2.2.1. Preprocessing

The preprocessing done to all the images was the following. First, all images were registered to the MNI512 template. An affine transformation was applied to the follow-up image, while for the basal image, the concatenation between two affine transformations, one from basal to follow-up scans and the one from follow-up scans to the MNI512 template, was applied. ANTs (Avants et al., 2009) with default linear interpolation was used for this purpose. Later, skull stripping was applied with HD-BET (Isensee et al., 2019), and finally, the images were normalized in the range [0–1].

#### 2.2.2. Proposed T1-w synthesis approach

The image generation architecture is based on the pix2pix architecture (Isola et al., 2017) which is a conditional GAN architecture where the network learn the mapping from the input to the output image as well as the loss function to train this mapping. Similarly to GANs, pix2pix architecture consists of a generator and a discriminator. During the training process, the generator tries to generate realistic samples in order to fool the discriminator while the discriminator tries to distinguish between real and synthetic samples (Xin et al., 2020).

**Figure 1:**
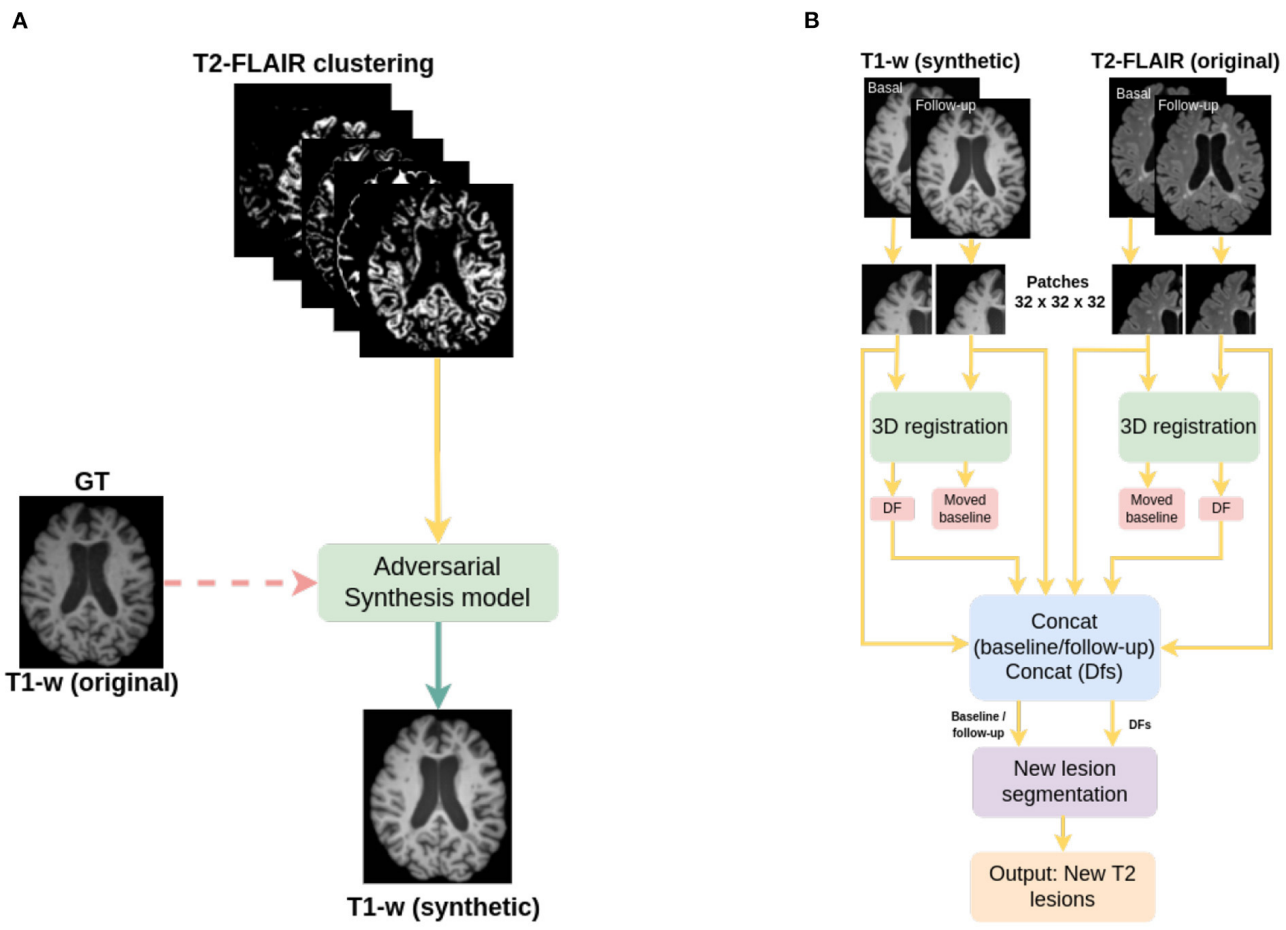
Pipelines used in this work; **(A)** pipeline for the synthesis of T1-w images, and **(B)** pipeline for new T2-w lesion detection in longitudinal analysis. The dashed line in **(A)** indicates that original T1-w images are used only in the training of the synthesizer.

A semantic image clustering of the T2-FLAIR image, which was obtained with the FSL FAST algorithm (Zhang et al., 2001; Jenkinson et al., 2012), together with its T1-w intensity pair as ground truth (Figure 1A) is used as input to the adversarial network. A different number of image clusters obtained using FSL FAST are considered in our experimental evaluation. We consider a minimum of 3 clusters corresponding to gray matter, white matter and cerebrospinal fluid (CSF), 5 clusters corresponding to gray matter, white matter, CSF and two partial volumes of the border between the tissues, and finally 7 and 9 clusters. These last clusterings of the image do not have a biological meaning but are considered here to study the impact on the synthesis model when smaller intensity clusters are used to perform the intensity mapping between modalities.

**Figure 2:**
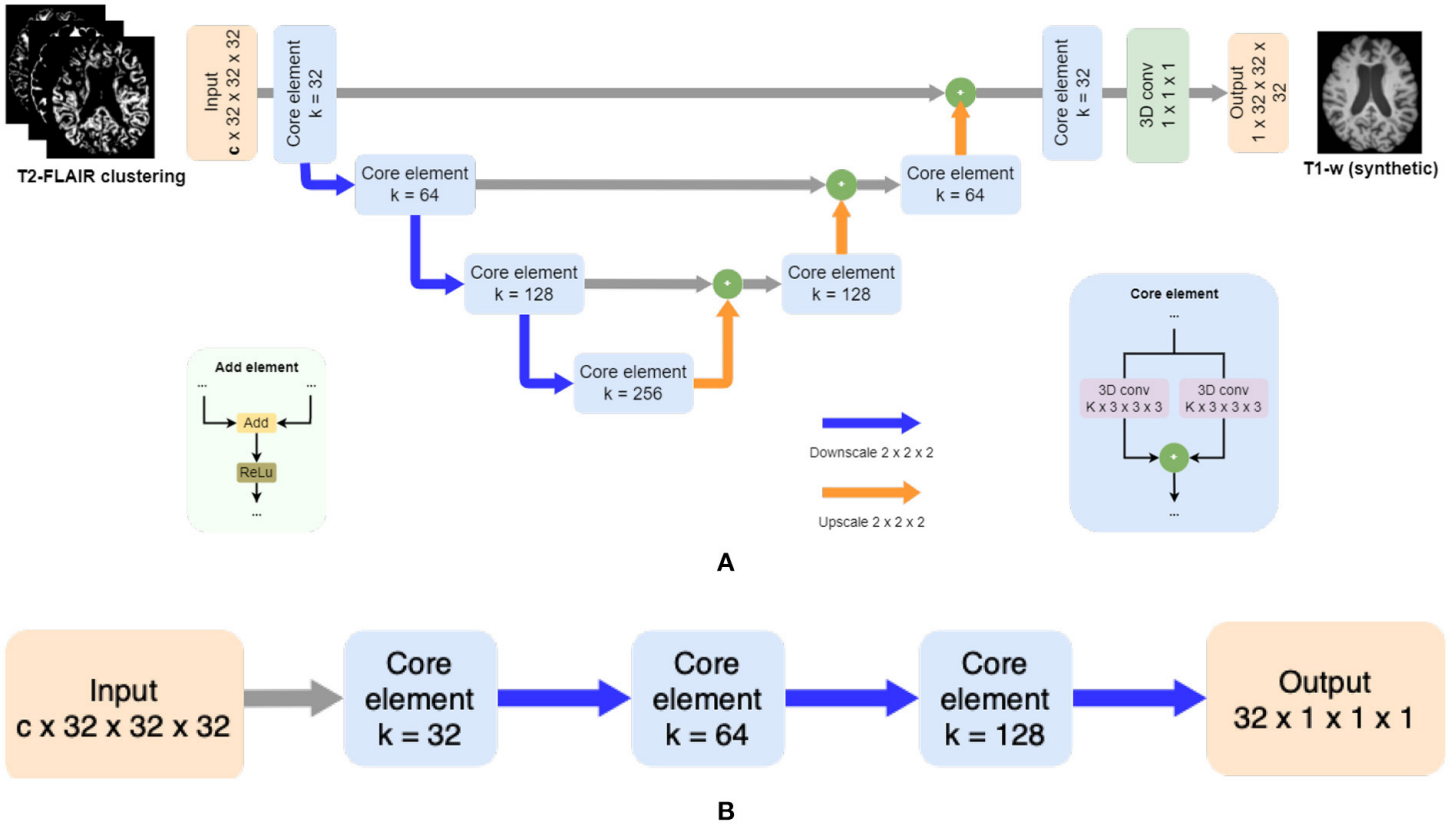
Adversarial synthesis model which takes as input the T2-FLAIR image clustering together with a T1-w intensity image as ground truth and generates a T1-w synthetic image as output. Core element modules in both architectures are composed of residual layers as described in He et al. (2016) with two convolutional layers (k = 3 x 3 x 3), followed by batch normalization, and finally a pooling layer by striding (*k* = 2 x 2 x 2). **(A)** The generator is a patchwise 3D encoder/decoder architecture where c is the number of clusters. Merged layers are implemented using summation instead of concatenation, using added element modules. **(B)** The discriminator is a patchwise 3D decoder architecture.

From each cluster volume and the T1-w image, patches of 32 x 32 x 32 are extracted and used as inputFrontiFron to the generator, which is a 3D ResUNet architecture of 8 blocks (Figure 2A), in essence a U-Net with residual layers. The UNet architecture (Ronneberger et al., 2015) is widely used in medical imaging due to its ability of capturing context through the extraction of high and low-level features and enable precise location. Adding residual connections allows merging feature maps from higher resolution layers with deconvolved maps to preserve localization details and improve back-propagation (He et al., 2016). Distinct from the original UNet architecture, which uses skip connections implemented with concatenations, we use summations to reduce the model complexity (Guerrero et al., 2018). After each residual layer in the downscaling path, pooling is applied. The discriminator is a ResNet with 4 blocks (Figure 2B), where the residual blocks are followed by pooling. Labels smoothing is used during the training of the model to improve the generalization and prevent the network to become over-confident about its prediction, therefore improving the accuracy (Müller et al., 2019).

Both the generator and discriminator have residual layers. Proposed by He et al. (2016), residual architectures facilitate the training of deeper networks, making them easier to optimize, and helping to improve the accuracy. Each block consists of two convolutions followed by batch normalization. The size of the kernel for the convolutions inside the residual blocks is 3 x 3 x 3. The pooling layers are implemented by striding with a kernel size of 2 x 2 x 2.

#### 2.2.3. New T2 lesion detection algorithm

The detection of new T2 lesions in longitudinal images is performed using the approach of Salem et al. (2020). It consists of a fully convolutional network (FCNN) that accounts for two 3D architectures: first registration and then segmentation, which are trained end-to-end. The inputs to the FCNN are the basal and follow-up images, while the output is a new T2 lesion segmentation mask (Figure 1B).

The network consists of two architectures: the first one is a 3D U-Net for registration where for each input modality, the architecture learns the deformation fields and nonlinearly register the baseline image to the follow-up image. A second architecture, a 3D U-net, performs the final detection and segments the new T2-w lesions. Gradient descent is used as the optimizer and the network simultaneously learns both deformation fields and the new T2-w lesion segments. The loss function of the registration architecture is an unsupervised loss function (Balakrishnan et al., 2019) which has two components: one that penalizes differences in appearance and a second one that penalizes local spatial variation. For the segmentation architecture, the well known cross-entropy loss function is used. The network was trained using 3D patches of 32 x 32 x 32 with a step size of 16 x 16 x 16 extracted from both baseline and follow-up images. Adam was used as optimizer.

In the original work, Salem et al. (2020), the input modalities were T1-w, T2-w, PD-w, and T2-FLAIR. In this work, we modified them to be only T2-FLAIR (referred to FLAIR-only) or T2-FLAIR and T1-w images (referred to T2-FLAIR + T1). The aim of this work is to evaluate the performance of the approach when using the synthetic T1 images generated as explained in the previous subsection.

### 2.3. Experimental evaluation

Three different experiments were performed in this study. First, we evaluated the image synthesis and determined which number of partial volumes improves the performance of the new T2 lesion detection algorithm.

**Figure 3:**
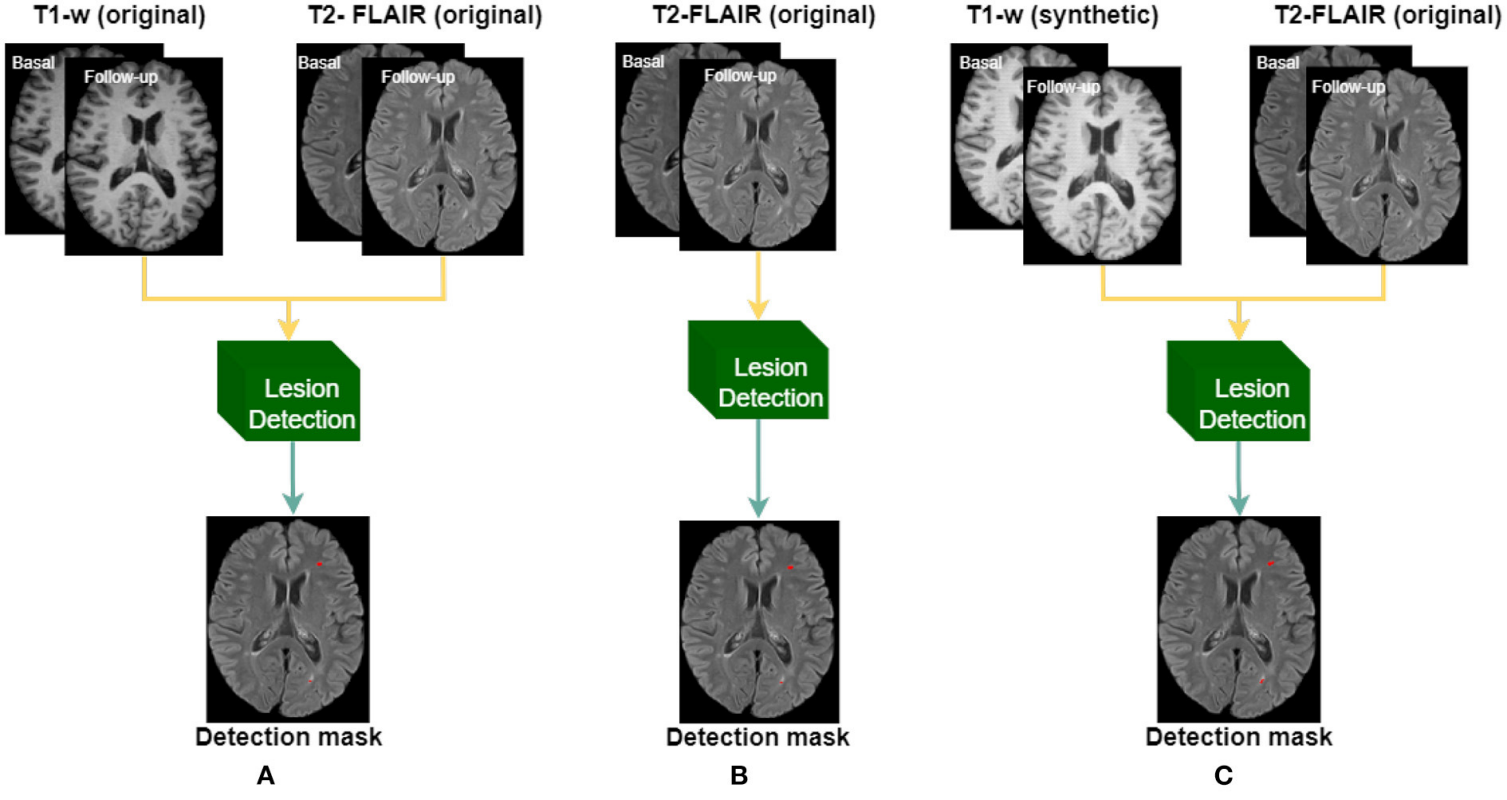
Three different models are trained according to the input images: **(A)** original T1-w and T2-FLAIR images, **(B)** using only original T2-FLAIR images, and **(C)** using synthetically obtained T1-w images (from the original T2-FLAIR images) along with the original T2-FLAIR images.

Subsequently, using the in-house dataset, we compared the performance of using T1-w synthetic images for the lesion detection against two different models trained with original images, as shown in Figure 3, and described as:

- Baseline: model trained using original T2-FLAIR and T1-w images.
- FLAIR-only: model trained using only original T2-FLAIR images.
- Synthetic: model trained using original T2-FLAIR original images and synthetic T1-w images, obtained from the original T2-FLAIR images.

Finally, we also evaluated our image synthesis and lesion detection proposal using the data from the international MSSEG-2 challenge (Commowick et al., 2021), showing the obtained performance when using FLAIR-only and when adding the generated T1-w images.

#### 2.3.1. Evaluation metrics for image quality

The quality of the images is evaluated locally measuring the voxel-wise intensity differences between a real image, *y*, and its approximation, $\bar{y}$, using the median absolute error (MAE) expressed as Equation (1). While the more similar images *y* and $\bar{y}$ are, the lower the MAE.


(1) $$\begin{array}{l} MAE\left(y,\bar{y}\right)=median\;|y-\bar{y}| \end{array}$$

For a global evaluation, we use the structural similarity index metric (SSIM) proposed by Wang et al. (2004) and defined in Equation (2), which accounts for variations in luminance, contrast, and structure correlation, and has been found to correlate with the quality of perception of the human visual system (Hore and Ziou, 2010). It is defined as:


(2) $$\begin{array}{l} SSIM\left(y,ỹ\right)=\frac{2\mu_{y}\mu_{ỹ}+c_{1}}{\mu_{y}^{2}+\mu_{ỹ}^{2}+c_{1}}\cdot \frac{2\sigma_{y}\sigma_{ỹ}+c_{2}}{\sigma_{y}^{2}+\sigma_{ỹ}^{2}+c_{2}}\cdot \frac{cov\left(y,ỹ\right)+c_{3}}{\sigma_{y}\sigma_{ỹ}+c_{3}}, \end{array}$$

where μ denote the mean and σ is the standard deviation values of the luminance of the images, *cov*(*y*, ỹ) is the covariance between *y* and ỹ, and *c*_*i*_ is a constant that is used to avoid a null denominator (Hore and Ziou, 2010). The SSIM values range within zero and one, where zero indicates null similarity and one indicates total similarity.

#### 2.3.2. Evaluation metrics for new T2 lesions detection performance

To evaluate the performance of the different trained models in the new T2 lesion detection algorithms, we use sensitivity, false discovery rate, and precision between the manual lesion annotation and the output segmentation mask. The sensitivity is defined as:


(3) $$\begin{array}{l} Sensitivity=\frac{TP}{TP+FN} \end{array}$$

where *TP* and *FN* denote the number of correctly and missed lesion region candidates, respectively. In terms of detection, a lesion is considered *TP* if there is one voxel overlapping (Cabezas et al., 2016; Salem et al., 2018, 2020). The false discovery rate is:


(4) $$\begin{array}{l} FDR=\frac{FP}{FP+TP} \end{array}$$

where *FP* denote the number of incorrectly classified lesion regions as positive. The precision is defined as:


(5) $$\begin{array}{l} Precision=\frac{TP}{TP+FP} \end{array}$$

where *TP* and *FP* denote the numbers of correctly and miss classified lesion region candidates, respectively.

#### 2.3.3. Statistical analysis

For each of the performance metrics of the detection of new T2 lesions, we applied the pairwise non-parametric Wilcoxon signed-rank test (two-sided) (Woolson, 2007), to assess the hypothesis of similar distributions between the different pairs of approaches. The results were considered significant for (*p* < 0.05).

## 3. Experimental results

To train and test the required models, we used the two subset configurations already available from the Vall d'Hebron Hospital. Set A included 101 patients, including 38 patients with new T2 lesions, and set B included 35 patients, all of whom had new T2 lesions. For the synthesis of T1-w images, set A was used for training, and set B was used for testing. Similarly, for the new T2 lesion detection models, the images from the 38 patients with new T2 lesions of set A were used for training, while the images from set B were used for testing (notice that for the model trained with synthetic images, the synthetic version of the images from set A were also computed).

We obtained the synthesized T1-w images using four different number of clusters of the T2-FLAIR image: 3, 5, 7, and 9 clusters. Table 1 shows the results of each case according to the similarity with the original image. For the inference of the new T2 lesion detection, voxels with ≥ 0.5 probability of being a lesion are taken as part of a lesion, while a lesion has a minimum of three neighboring voxels.

**Table 1:** Similarity between images and performance of the lesion detection algorithm when using 3, 5, 7, and 9 clusters in the synthesis of T1-w images.

	**Similarity**		**Detection**		
**Modalities**	**SSIM**	**MAE**	**Sensitivity**	**FDR**	**Precision**
T2-FLAIR + T1S (3c)	0.89 ± 0.07	0.11 ± 0.05	0.51 ± 0.38	0.07 ± 0.17	0.69 ± 0.42
T2-FLAIR + T1S (5c)	0.91 ± 0.07⋆	0.09 ± 0.05⋆	0.73 ± 0.31⋆	0.11 ± 0.20	0.83 ± 0.29⋆
T2-FLAIR + T1S (7c)	0.90 ± 0.07	0.10 ± 0.05	0.81 ± 0.23▿	0.14 ± 0.19	0.86 ± 0.19▿
T2-FLAIR + T1S (9c)	0.90 ± 0.07	0.09 ± 0.05 †	0.77 ± 0.30 †	0.25 ± 0.27 ◇	0.73 ± 0.29

Significant differences in metrics between 5c and 3c are marked with ⋆, differences between 7c and 3c are marked with ▿, while differences between 9c and 3c are marked with †. Results of FDR for 9c are significantly lower with respect to the other 3 approaches (marked with ◇).

**Figure 4:**
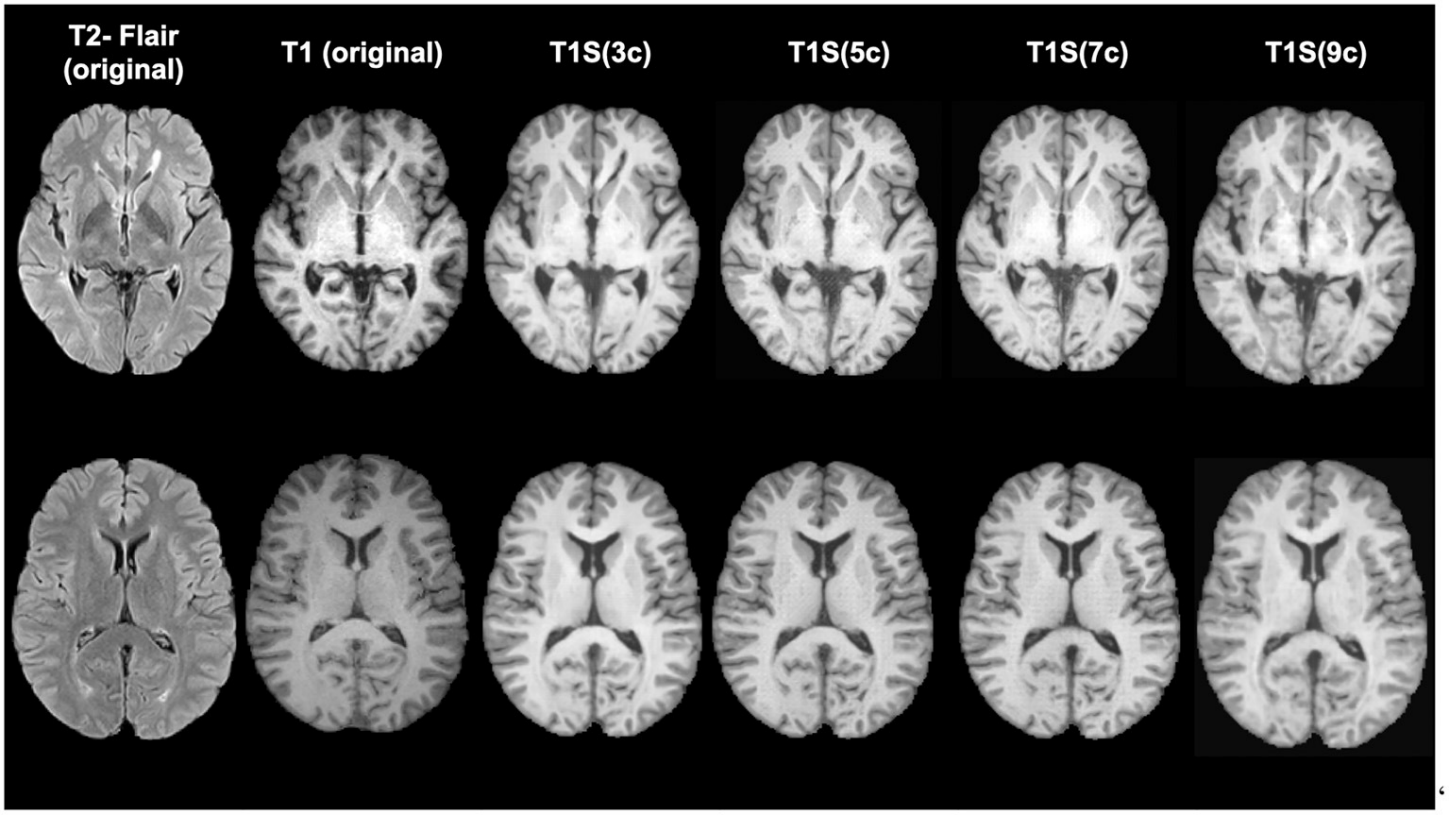
Examples of original and synthetically obtained images. The first column shows the original T2-FLAIR image, while the second column shows the original T1-w image. The following columns show the T1-w images obtained from the T2-FLAIR image using a different number of clusters (3, 5, 7, and 9).

According to the similarity measures, the most similar image was obtained when using 5 clusters. Differences according to SSIM are small, while using MAE the performance of using 5 and 9 clusters are significantly different (*p* < 0.05) than when using 3 clusters. This difference in behavior of the measures shows the benefit of comparing the similarity between images both globally and locally. Figure 4 shows a qualitative example of each case, showing a high global similarity with respect to the ground truth, although there are discrepancies, mainly in the borders of the tissues, which are captured by the local similarity. Although the adversarial network exhibits common artifacts such as the intensity shift, they were more visible when using the approach with 3 clusters. On the contrary, when using 5, 7 and 9 clusters, axial slices generated tend to preserve better delineation of some structures.

Table 1 also shows the detection inferences computed with a fixed voxel probability threshold ≥ 0.5. Note that when using T1S (7c), higher sensitivity and precision were obtained. To make these values more comparable, we inferred the detection using a threshold ≥ 0.3 for T1S (3c), T1S (5c) and T1S (9c) in an attempt to reach a similar operating point to that of the approach using T1S (7c). Under these conditions, T1S (3c) reached a sensitivity of 0.67 ± 0.33 with 0.14 ± 0.24 FDR, T1S (5c) increased the sensitivity to 0.78 ± 0.29 but with an FDR of 0.27 ± 0.3, while T1S (9c) reached a sensitivity of 0.79 ± 0.34 with 0.30 ± 0.26 FDR. Considering all these results, we can see that although all models were able to detect lesions, the best trade-off with the different detection measures was obtained when using 7 clusters in the synthesis of the T1-w sequence. Notice that images generated with 7c were not showing the best overall quality measurements but provided better feature information to improve the MS lesion detection.

Applying the synthesis based on 7 clusters (7c), we also evaluated the use of the synthetic T1-w images on the performance of the detection, using the 3 different approaches seen in Figure 3. Table 2 shows the obtained results. When using the original images, the sensitivity was 0.75 ± 0.29 at FDR of 0.09 ± 0.18, while when using only T2-FLAIR images as input the values were 0.63 ± 0.37 and 0.14 ± 0.24, respectively. When using the T2-FLAIR images along with the T1-w images synthesized from the same T2-FLAIR image, as an input, the sensitivity increased to 0.81 ± 0.23, without increasing the FDR with respect to the model using only T2-FLAIR images. The increase in sensitivity was significant with respect to the other models (*p* < 0.05). The precision between models showed that when using only T2-FLAIR images the performance was significantly lower (*p* < 0.05) than when using T1-w images, either real or synthesized. Comparing the use of both kinds of images, the results were similar.

**Table 2:** New T2 lesion detection performance evaluation using the models shown in Figure 3.

**Modalities**	**Sensitivity**	**FDR**	**Precision**
**Results with original images**			
T2-FLAIR + T1(Baseline)	0.75 ± 0.29	0.09 ± 0.18	0.85 ± 0.27
T2-FLAIR (FLAIR-only)	0.63 ± 0.37	0.14 ± 0.24	0.71 ± 0.38
**Results with synthetic T1**			
T2-FLAIR + T1S (7c)	0.81 ± 0.23 ⋆ ▿	0.14 ± 0.19	0.86 ± 0.19▿

Significant differences of the T2-FLAIR + T1S (7c) model w.r.t the Baseline and FLAIR-only models are marked with ⋆ and ▿, respectively.

### 3.1. Results using the MSSEG-2 dataset

In this experiment, we used our adversarial synthesis model trained with the in-house dataset to generate T1-w images for all the cases of the international MSSEG-2 challenge, where only T2-FLAIR images were available (Commowick et al., 2021). We compared the performance of the MS lesion detection approach using only the T2-FLAIR images [original VICOROB submission to the challenge using Salem et al. (2020) with only T2-FLAIR images] vs. the model trained using both T2-FLAIR and T1-w synthetic images. Notice that all the MSSEG-2 training dataset was used to train both models, while the evaluation was done directly using the MSSEG-2 testing set, including both the active and stable cases.

The obtained results are illustrated in Table 3, where the two approaches are compared with some of the best pipelines participating in the challenge. Table 3 illustrates also the agreement of the approaches with the different expert raters. Interestingly, the performance of the model when using T1-w synthetic images was higher than the model using only T2-FLAIR images. For the active patients, we obtained an improvement in terms of sensitivity and precision of 0.12 and 0.2, respectively, while also reducing the FDR. Notice that the accuracy of the model was similar to that of some of the top participants in the challenge (MEDIARE_*B*_, EMPENN and SNAC, see the MSSEG-2 challenge webpage for details of the participants), yielding also a performance that was comparable in terms of sensitivity to those of the human raters. Regarding the stable patients, where no new lesions were present, we observed a reduction in the total number of FP obtained and in the number of cases with FPs (11% of the 28 stable cases). Furthermore, it should be noted that our synthesis model was trained directly using the in-house dataset and only using images from a Siemens machine. This shows a capability of the model to adapt the source knowledge into the target domain of the challenge where data from different MRI scanners were available, producing T1-w images which indeed could be used to improve MS lesion detection.

**Table 3:** Results of the MSSEG-2 challenge 2021.

**MSSEG-2 challenge**	**Active patients**			**Stable patients**
	**Sensitivity**	**FDR**	**Precision**	**N° of cases with FP (%)**
Expert 1	0.71 ± 0.38	019 ± 0.31	0.72 ± 0.38	1 (4%)
Expert 2	0.61 ± 0.37	0.13 ± 0.21	0.68 ± 0.39	3 (11%)
Expert 3	0.61 ± 0.37	0.13 ± 0.23	0.69 ± 0.40	0 (0%)
Expert 4	0.47 ± 0.39	0.06 ± 0.19	0.66 ± 0.46	1 (4%)
MEDIARE_*B*_	0.69 ± 0.40	0.39 ± 0.34	0.49 ± 0.36	10 (36%)
EMPENN	0.59 ± 0.32	0.33 ± 0.32	0.51 ± 0.36	8 (29%)
SNAC	0.66 ± 0.40	0.39 ± 0.33	0.49 ± 0.35	4 (14%)
VICOROB (FLAIR-only)	0.50 ± 0.39	0.43 ± 0.34	0.35 ± 0.32	6 (23%)
VICOROB (FLAIR + T1S)	0.62 ± 0.39	0.39 ± 0.38	0.55 ± 0.39	3 (11%)

Testing set composed by 60 patients: active patients *n* = 32, stable patients *n* = 28. Sensitivity, FPR and precision are shown for active patients, while the number of cases that presented FPs are provided for stable cases.

## 4. Discussion

In this study, we investigated the usefulness of synthetic T1-w images in a longitudinal lesion detection pipeline. Starting from single T2-FLAIR images, we propose obtaining synthesized T1-w images that are subsequently used as an additional image modality to look for new abnormalities in the longitudinal analysis of the brain. Experiments show that although strong structural differences exist between T2-FLAIR and T1-w images, given the contrast difference between the two modalities, realistic T1-w images were able to be produced. In addition, the results show that adding the synthetic images to T2-FLAIR images in the detection pipeline provides new and reliable information that helps obtain better detection.

Our approach for generating T1-w images relies on intensity clustering of the T2-FLAIR images. The obtained clusters allow us to guide intensity information during the generation process. We have shown that images using more than 3 clusters are more similar to the original T1-w images. Most likely, the use of a few clusters does not account for the inherent partial volumes of MR images, while using more clusters allows better mapping of the partial volumes.

**Figure 5:**
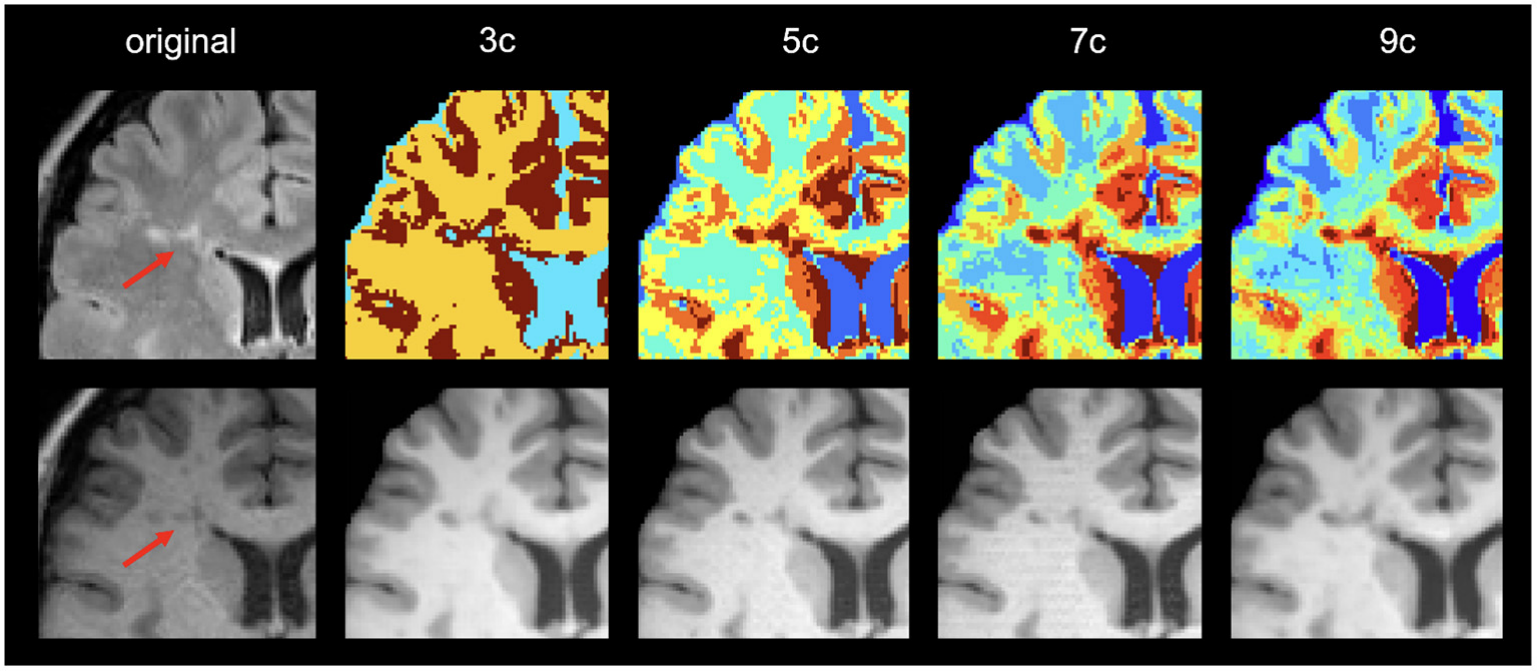
Example of image generations in a lesion area. First column shows the original T2-FLAIR and T1 image. The rest of the columns show the clustering result and the corresponding generated image using different numbers of clusters (3, 5, 7, and 9, respectively).

Regarding the lesion detection process, the best results were obtained when using 7 clusters. We observed that using more than 3 clusters allowed us to obtain additional information from the lesion areas that turns out to help in the lesion detection process. Note that the main goal of the synthesis is to provide images with complementary information to the network to improve lesion detection rather than produce high-quality synthetic images. Interestingly, we noticed that in the lesion areas, the model using 9 clusters tended to resemble too much the original T2-FLAIR cluster intensities in the generated T1 images, forcing an intensity mapping that deviates from the intensities present in the original T1. This can be seen in the example shown in Figure 5, where the generated image using 9 clusters produces more hypointense voxels in the lesion area than in the original T1 due to the larger number of clusters used and the intensity mapping learned from the model.

Comparing the detection performance when using only T2-FLAIR images vs. adding synthetic T1-w images, we found that there was a statistically significant difference in sensitivity between the two models. This indicates that the addition of T1-w synthetic images provides meaningful and additional information for the detection of the lesions. In contrast, the performance when using original T1-w images or synthetic images is similar, although we obtained slightly better results with the synthetic images. Our hypothesis is that in image synthesis, what is learned during training are the most predominant features of a T1-w image that can be extracted from a T2-FLAIR modality. These features may be related to the lesions, and therefore, the sensitivity during detection could improve. This may also be related to the number of clusters used. When using 5 clusters, we obtained more similar images than using 7 clusters, although the best performance for lesion detection was obtained when using the synthetic images from 7 clusters.

There is one limitation of this work that should be mentioned. All the images used in the study to train the synthesis model were taken from the same scanner, which was a Siemens Tim Trio 3T. Although the experiments done using the MSSEG-2 Challenge showed the capability of the synthesized images to improve the MS lesion detection even when using images from different MRI scanners (Siemens, Philips and GE), further investigations should be done in this line. As a future work, we plan to evaluate more exhaustively our synthesis approach when using images from different MRI scanners, analyzing not only the impact on the image generation and on the lesion detection performance, but also its applicability as an image standardization procedure. Furthermore, it could be very interesting to extend the study using more advanced synthesis models such as cycleGAN (Zhu et al., 2017) or Hi-Net (Zhou et al., 2020), which could in turn improve the generalization and the performance of the MS lesion detection approaches.

In conclusion, the results shown in this work demonstrate that the inclusion of synthetic images can support the lack of data. Specifically, we have seen how the inclusion of synthetic T1-w images on the lesion detection models helped to improve the overall performance. Our approach could benefit the clinical acquisition of MRI sequences, helping to reduce time and costs. Moreover, synthetic images could also be used instead of the original images to homogenize the contrast of the different acquisitions.

## Data availability statement

The datasets presented in this article are not readily available because the dataset used in this work is an in-house dataset from the Vall d'Hebron Hospital (Barcelona, Spain) that includes T1-w and FLAIR images from 136 MS patients. Informed consent was obtained from each participant before enrolment in the study. The agreement done for sharing the data restricts the usability of the entities participating in this research study. Requests to access the datasets should be directed to xavier.llado@udg.edu.

## Ethics statement

The studies involving human participants were reviewed and approved by Vall d'Hebron Hospital (Barcelona, Spain) Research and Ethics Committee. The patients/participants provided their written informed consent to participate in this study.

## Author contributions

LV, AC, MS, SV, AO, and XL contributed to the conception and design of the study. ÀR organized the database and provided clinical information. All authors contributed to manuscript revision, read, and approved the submitted version.

## Funding

AC holds an FPI grant from the Ministerio de Ciencia, Innovación y Universidades with reference number PRE2018-083507. This work has been supported by DPI2020-114769RB-I00 from the Ministerio de Ciencia, Innovación y Universidades. The authors gratefully acknowledge the support of the NVIDIA Corporation with their donation of the TITAN X GPU used in this research. This work has been also supported by ICREA Academia Program.

## Conflict of interest

Author SV was employed by company Tensor Medical. The remaining authors declare that the research was conducted in the absence of any commercial or financial relationships that could be construed as a potential conflict of interest.

## Publisher's note

All claims expressed in this article are solely those of the authors and do not necessarily represent those of their affiliated organizations, or those of the publisher, the editors and the reviewers. Any product that may be evaluated in this article, or claim that may be made by its manufacturer, is not guaranteed or endorsed by the publisher.
